# Improving Constipation in Critical Care Patients: A Single-Centre Prospective Cohort Closed-Loop Clinical Audit

**DOI:** 10.7759/cureus.101232

**Published:** 2026-01-10

**Authors:** Naining Xu, Chian Chyn Khoo

**Affiliations:** 1 Intensive Care Unit, Royal Liverpool University Hospital, Liverpool, GBR; 2 Critical Care Medicine, Royal Liverpool University Hospital, Liverpool, GBR

**Keywords:** bowel protocol, clinical audit, constipation, critical care, icu (intensive care unit)

## Abstract

Background

Constipation affects many critically ill patients and may lead to discomfort, prolonged hospital stay, or mortality. Standardised bowel protocols are recommended to improve patient care. This audit evaluates the adherence to the bowel protocol at the Intensive Care Unit (ICU) at Royal Liverpool University Hospital, before and after educational interventions.

Methods

Adult ICU patients aged 18 and above were included. Patients who had post-gastroenterological surgery, hepatic encephalopathy, bowel obstruction/ischaemia, or spinal cord injury were excluded from this study. Data were collected from paper charts and the internal hospital electronic system. Two four-week cycles of data were collected (20/05/2024-16/06/2024, 10/07/2024-07/08/2024), with the intervention of the delivery of educational sessions and posters implemented between the two cycles.

Results

In the first cycle, 50 patients were assessed: 14 medical and 36 surgical. Enteral feeding within 48 hours occurred in 100% of medical and 88.9% of surgical patients. Senna and docusate were prescribed in 50% of medical and 25% of surgical patients on admission. Bowel movements within 48 hours occurred in 64.3% of medical and 36.1% of surgical patients (p=0.113 when comparing within the first cycle). In the second cycle, 48 patients were included: 10 medical and 38 surgical. Senna and docusate were prescribed in 90% of medical patients, with 90% achieving bowel movements within 48 hours. Surgical patients showed lower adherence, with 13.2% prescribed senna and docusate and 21.1% opening bowels within 48 hours. Within the second cycle, the difference in the onset of bowel motion between medical and surgical patients was statistically significant (p=0.0001). However, no statistical changes were found comparing the first and second cycles in medical and surgical patients prospectively.

Conclusion

The outcome of this audit suggests, but does not confirm, that educational interventions may positively impact the adherence to bowel protocol in ICU patients. Surgical patients demonstrated limited change due to post-operative care plans determined by parent teams. Ongoing education is essential for optimising the compliance of bowel protocol in the ICU.

## Introduction

Constipation is a common complication which occurs in critically ill patients [1]. It can be defined based on the infrequency of passage of stool, typically three times or less per week, or based on the consistency of stool [2]. Some literatures also define constipation based on the symptoms associated with it, including prolonged straining or the sensation of incomplete evacuation [3]. Due to the lack of a universal definition of constipation, the published estimated rate of constipation ranges widely. It is noted that 24-83% of patients in critical care units experience constipation [4,5]. Several factors contribute to this, including electrolyte disturbances, intestinal hypoperfusion secondary to shock, prolonged immobility, post-operative related dietary restrictions, insufficient intravenous or oral fluid administration and the use of opioid medications [6-8].

Failure to manage or address such issues can result in patients’ discomfort and prolonged hospital stay. If left unrecognised and untreated, constipation could potentially lead to serious consequences, including mortality [7]. It is therefore crucial to prevent and manage constipation proactively in patients who are admitted to the critical care unit.

This audit aims to evaluate and improve the current adherence to the pre-existing bowel protocol at Royal Liverpool University Hospital. The specific objectives of this audit are, firstly, to assess the adherence of the bowel protocol in critical care patients; secondly, to implement educational interventions to improve compliance with the protocol; and finally, to re-evaluate the changes made in the rate of adherence to the protocol following these interventions.

## Materials and methods

This study was conducted at the Intensive Care Unit (ICU) at Royal Liverpool University Hospital, Liverpool, United Kingdom. This audit was approved by the clinical audit department at the Royal Liverpool University Hospital in 2024. Data collection took place from May 2024 to August 2024.

**Figure 1:**
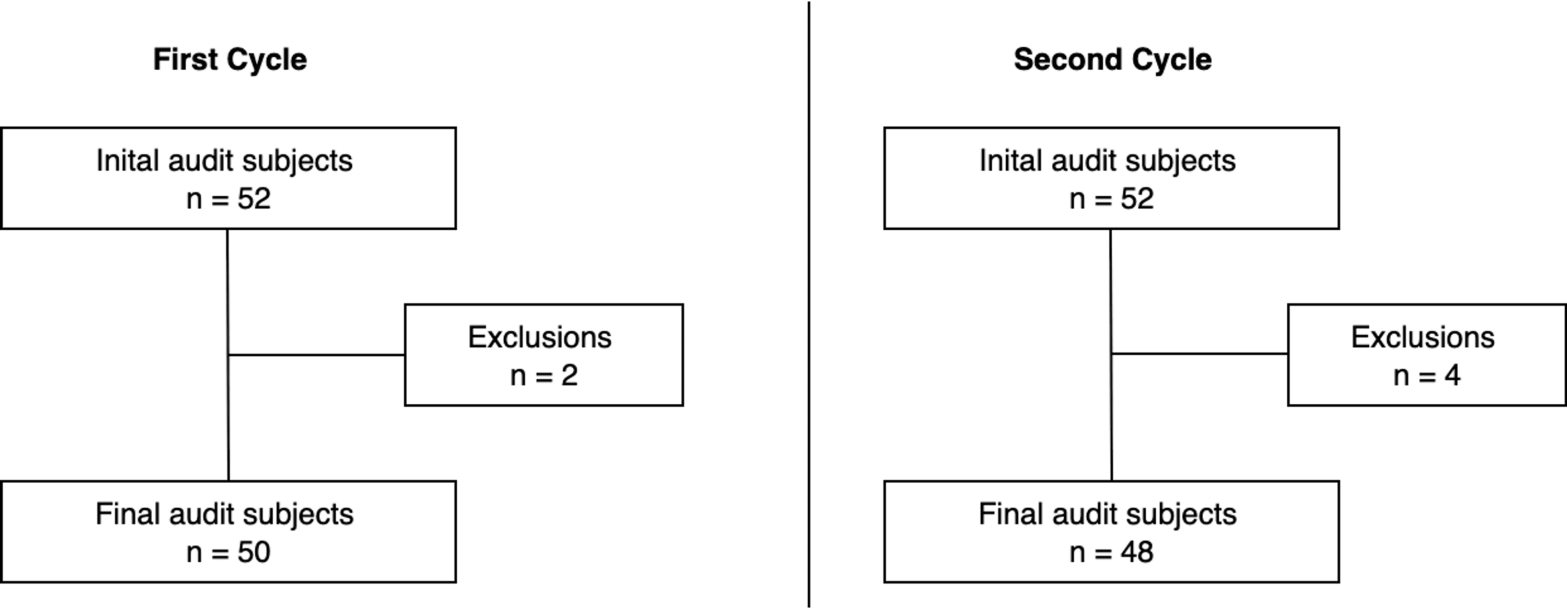
Inclusion subjects for first and second cycle of data collection

This prospective cohort study included all adult patients aged 18 or above who were admitted to the ICU. Patients who have the following conditions were excluded from the audit: post-gastroenterological surgery, bowel obstruction/ischaemia, hepatic encephalopathy and spinal cord injury (SCI) (Figure 1). Bowel obstruction/ischaemia and bowel surgeries were excluded due to the natural slower intestinal transit related to the intentional slower build-up of oral intake and post-operative ileus [9]. Patients with SCI or hepatic encephalopathy were excluded as this study focuses on constipation resulting from the primary reason for ICU admission, and the pathophysiology of constipation in these conditions differs from that of the study population [10,11]. 

**Figure 2:**
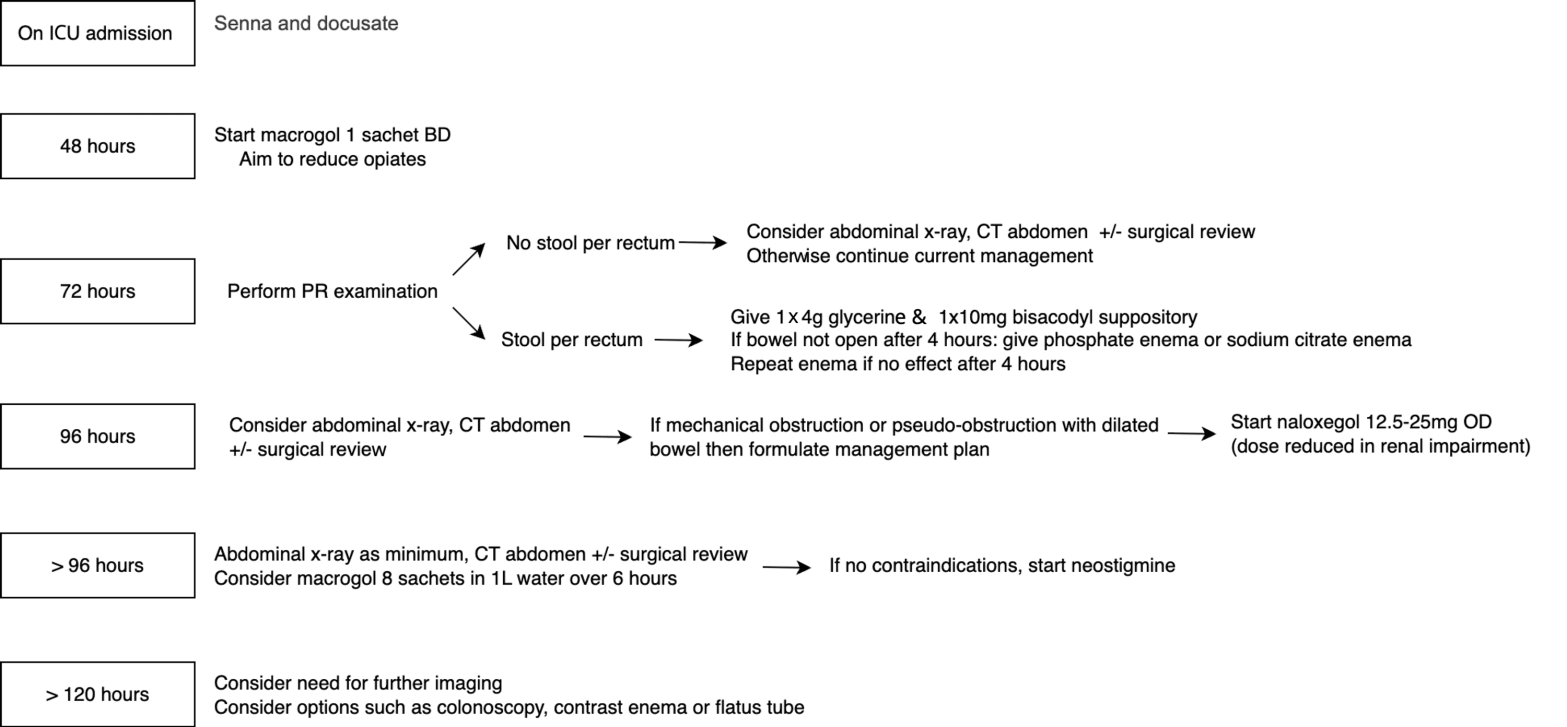
Bowel protocol in Intensive Care Unit (ICU) at Royal Liverpool University Hospital

The primary outcome measured was whether patients had a bowel motion at different time points following their admission: 48, 72, 96, 120 hours or beyond. A bowel chart was used for daily documentation of bowel motion for all patients. Secondary data were also recorded according to the pre-existing bowel protocol, which dictates whether various actions were performed at different time points (Figure 2).

The first round of data was collected over a four-week period, from 20/05/2024 to 16/06/2024. Following this, a three-week break was implemented during which educational sessions were conducted for the unit's healthcare staff, complemented by the display of educational posters throughout the unit. The second round of data collection took place over a further four-week period, from 10/07/2024 to 07/08/2024.

All data were gathered using ICU observation charts, drug charts, and the electronic inpatient system (dashboard). All data was collected by one person, and a standardised format was followed. Admissions were reviewed daily. To the author's knowledge, no missing data was encountered. The pre-existed bowel protocol was obtained from the hospital intranet (Figure 2).

The findings of this audit were subsequently presented at the local audit meeting in February 2025 to all consultant intensivists and all resident doctors who worked in the department at the time.

## Results

First cycle

In total, 50 subjects participated in the first cycle of data collection (Table 1). Fourteen (28%) were medical patients, while 36 (72%) were surgical patients. All medical patients were commenced on enteral feeding within 48 hours of admission to the ICU. Senna and docusate were prescribed for 50% of these medical patients, with nine out of 14 (64.3%) experiencing bowel movements within 48 hours.

**Table 1:** Summary of first cycle data collection PR: per rectum

Parameters (first cycle data collection)	Number of patients	Percentage	Number of patients	Percentage
	Medical		Surgical	
Enteral feeding within 48 hours	14/14	100%	32/36	88.9%
Senna & docusate on admission	7/14	50%	9/36	25%
At 48 hours				
Bowel opened	9/14	64.3%	13/36	36.1%
Macrogol	2/14	14.3%	2/36	5.6%
At 72 hours				
Bowel opened	11/14	78.6%	13/36	36.1%
PR examination	0/14	0%	1/36	2.8%
Glycerine suppositories	2/14	14.3%	1/36	2.8%
Bisacodyl suppositories	0/14	0%	0/36	0%
Phosphate/sodium citrate enema	0/14	0%	0/36	0%
Abdo examination	0/14	0%	36/36	100%
Abdo X-ray	0/14	0%	0/36	0%
Abdo CT	0/14	0%	0/36	0%
Surgical review	0/14	0%	36/36	100%
At 96 hours				
Bowel opened	11/14	78.6%	33/36	91.7%
Abdo X-ray	0/14	0%	0/36	0%
Naloxegol	0/14	0%	0/36	0%
Eight sachets of macrogol	0/14	0%	0/36	0%
Abdo CT	0/14	0%	1/36	2.8%
Surgical review	0/14	0%	36/36	100%
Neostigmine	0/14	0%	0/36	0%
At 120 hours or more				
Bowel opened	11/14	78.6%	35/36	97.2%
Abdo X-ray	0/14	0%	0/36	0%
Abdo CT	0/14	0%	1/36	2.8%
Surgical review	0/14	0%	36/36	100%
Gastro review	0/14	0%	0/36	0%
Eight sachets of macrogol	0/14	0%	0/36	0%
Colonoscopy	0/14	0%	0/36	0%
Contrast enema	0/14	0%	0/36	0%
Flatus tube	0/14	0%	0/36	0%

Additionally, two patients (14.3%) were given macrogol at the 48-hour mark and subsequently opened their bowels. By 72 hours, 11 out of 14 patients (78.6%) had bowel movements. Notably, no patients underwent a per rectum (PR) examination. Glycerine suppositories were used in two out of 14 cases (14.3%). Neither bisacodyl suppositories nor sodium citrate enemas were administered.

For the surgical patients, 32 out of 36 (88.9%) started enteral feeding within 48 hours. Only nine patients (25%) received senna and docusate. Thirteen out of 36 (36.1%) had bowel movements within the first 48 hours. Macrogol was given to two out of 36 patients (5.6%) at 48 hours. A PR examination was conducted on one patient (2.8%), and glycerine suppositories were used in one out of 36 patients (2.8%). There were no instances of bisacodyl suppositories or sodium citrate enemas being used. Patients were regularly assessed by their parent surgical team during their ICU stay. This included regular abdominal examinations.

Fisher's exact test was performed to compare the statistical significance of the rate of bowel motion at different time frames between the medical and surgical patients in the first cycle. At 48 hours, the rate of bowel motion in medical patients (64.3%) was higher compared to the surgical cohort (36.1%). However, this was statistically insignificant (p=0.113). At 72 hours, medical patients were significantly more likely to open their bowels (78.6% versus 36.1%, p=0.011). At 96 hours and 120 hours and beyond, no significant differences were found in the rate between medical and surgical patients (p=0.331, p=0.061, respectively).

Second cycle

Among the 48 subjects, 10 (20.8%) were medical patients, while 38 (79.2%) were surgical patients (Table 2). Of the medical patients, nine out of 10 (90%) began enteral feeding within 48 hours of admission. Senna and docusate were prescribed for 90% of these medical patients, and notably, nine out of 10 (90%) experienced bowel movements within the same timeframe. Additionally, two out of 10 patients (20%) had macrogol included in their medication charts. No patients underwent PR examinations. Glycerine suppositories, bisacodyl suppositories, or sodium citrate enemas were not given to any of the patients.

**Table 2:** Summary of second cycle data collection

Parameters (second cycle data collection)	Number of patients	Percentage	Number of patients	Percentage
	Medical		Surgical	
Enteral feeding within 48 hours	9/10	90%	31/38	81.6%
Senna & docusate on admission	9/10	90%	5/38	13.2%
At 48 hours				
Bowel opened	9/10	90%	8/38	21.1%
Macrogol	2/10	20%	0/38	0%
At 72 hours				
Bowel opened	9/10	90%	14/38	36.8%
PR examination	0/10	0%	0/38	0%
Glycerine suppositories	0/10	0%	0/38	0%
Bisacodyl suppositories	0/10	0%	0/38	0%
Phosphate/sodium citrate enema	0/10	0%	0/38	0%
Abdo examination	0/10	0%	38/38	100%
Abdo X-ray	0/10	0%	0/38	0%
Abdo CT	0/10	0%	1/38	2.6%
Surgical review	0/10	0%	38/38	100%
At 96 hours				
Bowel opened	9/10	90%	29/38	76.3%
Abdo X-ray	0/10	0%	0/38	0%
Naloxegol	0/10	0%	0/38	0%
Eight sachets of macrogol	0/10	0%	0/38	0%
Abdo CT	0/10	0%	2/38	5.2%
Surgical review	0/10	0%	38/38	100%
Neostigmine	0/10	0%	0/38	0%
At 120 hours or more				
Bowel opened	9/10	90%	33/38	86.8%
Abdo X-ray	0/10	0%	0/38	0%
Abdo CT	0/10	0%	3/38	7.9%
Surgical review	0/10	0%	38/38	100%
Gastro review	0/10	0%	0/38	0%
Eight sachets of macrogol	0/10	0%	0/38	0%
Colonoscopy	0/10	0%	0/38	0%
Contrast enema	0/10	0%	0/38	0%
Flatus tube	0/10	0%	0/38	0%

In the surgical cohort, 31 out of 38 patients (81.6%) were commenced on enteral feeding within 48 hours. Senna and docusate were prescribed for only five out of 38 patients (13.2%), and eight out of 38 (21.1%) had bowel movements within 48 hours. No patients received macrogol during this period. Similar to the medical cohort, glycerine suppositories, bisacodyl suppositories, and sodium citrate enemas were not used, and PR examinations were not conducted.

Fisher's exact test was again performed to compare the results between medical and surgical patients within this cycle. It was noted that at 48 hours, the difference between the rate of bowel opening in medical patients (90%) and surgical patients (21.1%) was significant (p=0.0001). At 72 hours, the difference was also significant (90% versus 36.8%, p=0.0038). At 96 hours and 120 hours and beyond, no significant differences were found in the data (p=0.664, p=1.00, respectively).

Overall

Statistical comparison was performed to evaluate the rate of bowel opening at different time frames between the first and second cycles. Fisher's exact test was also used due to the overall small sample size. No statistical difference was found between the two cycles in either the medical or surgical cohort (Table 3).

**Table 3:** Statistical significance in medical and surgical patients between first and second cycle

Bowel motion time frame	Medical patients (first vs. second cycle)	Surgical patients (first vs. second cycle)
At 48 hours	p=0.341	p=0.199
At 72 hours	p=0.615	p=1.000
At 96 hours	p=0.615	p=0.114
At 120 hours or more	p=0.615	p=0.200

## Discussion

**Figure 3:**
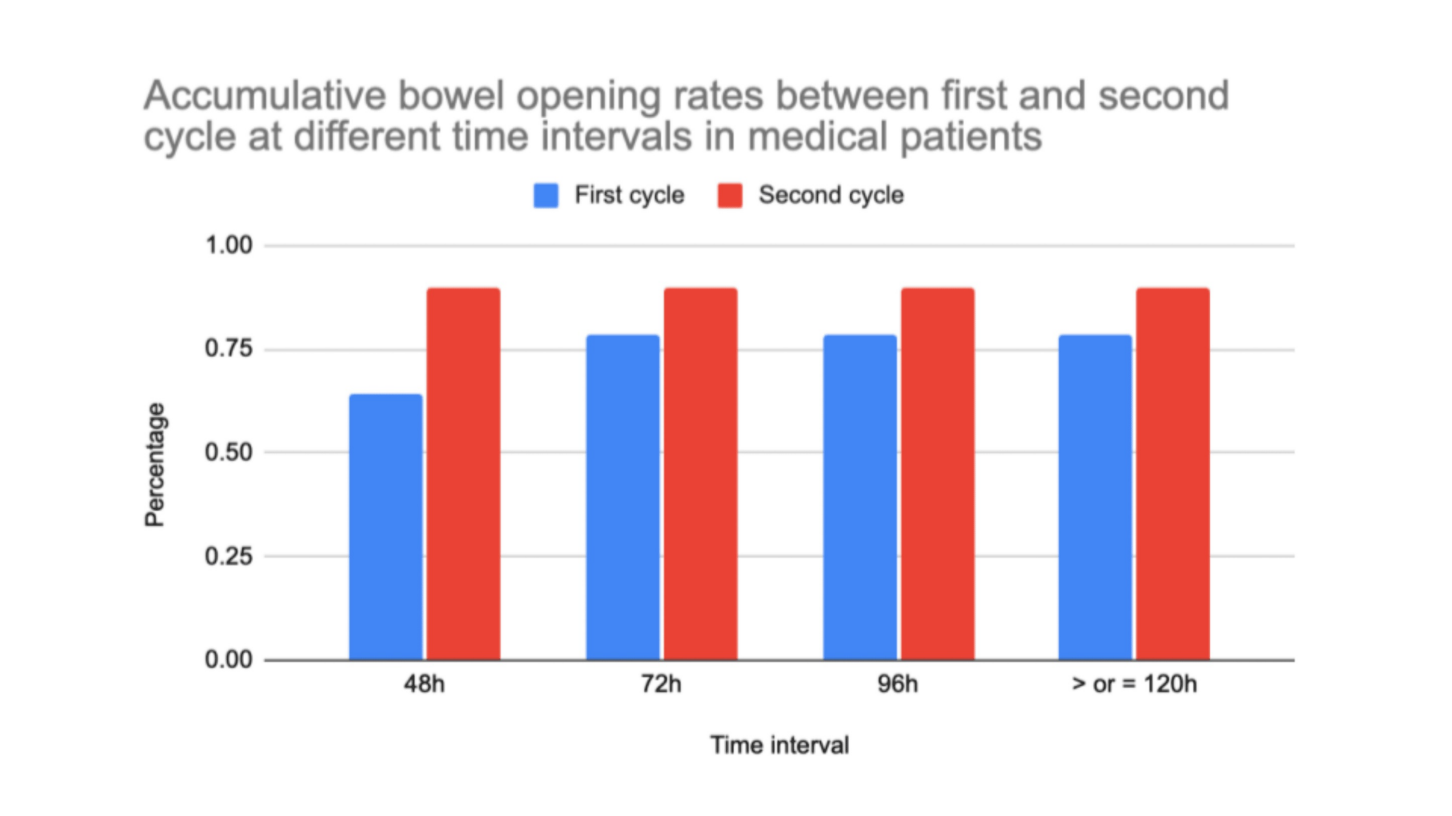
Accumulative bowel opening rates between first and second cycle at different time intervals in medical patients

**Figure 4:**
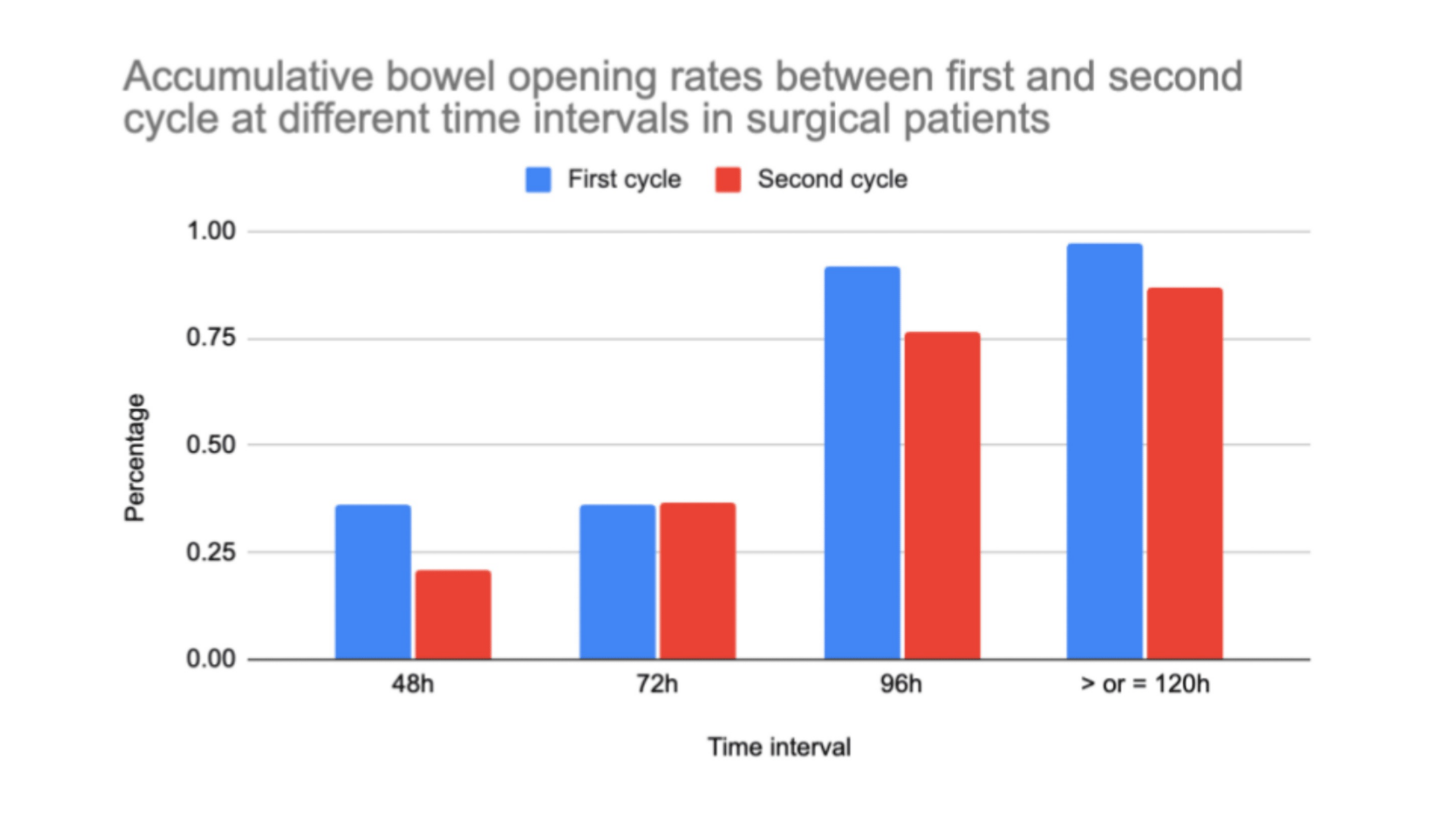
Accumulative bowel opening rates between first and second cycle at different time intervals in surgical patients

Overall, the outcome of this audit suggests a possible improvement in compliance with the bowel protocol from cycle one to cycle two, following educational interventions (Figures 3, 4). Although these changes were not statistically significant, Figure 3 and Figure 4 reflect a positive trend in bowel protocol adherence.

It is noted that the combination of senna and docusate was shown to improve constipation in the study by Patel et al., looking at bowel management in post-operative patients who underwent pelvic reconstructive surgery [12]. Senna acts as a stimulant laxative, which promotes intestinal peristalsis. Docusate belongs to the class of stool softeners, which allows water penetration into the stools [13]. Both agents have a relatively faster rate of action (8-12 hours), and due to their readily accessible nature, they are appropriate to be used as the first step in the protocol [14].

Although constipation is commonly defined as a period of difficulty or inability to pass stools for three times or less per week, pharmacological and preventative measures should be commenced at the time of ICU admission, according to the standardised hospital bowel protocol for best practices [15].

It is recommended to consider reducing the dose of opioids and commencing macrogol at 48 hours if patients have not opened their bowels. Opioids are often used in critical care settings, specifically for mechanical ventilation and for post-operative pain management. Moran et al. reported that approximately 80% of patients who require mechanical ventilation need regular opioids, and the use of opioids has been the mainstay of pain management in patients who underwent major operations [15,16]. The major side effect of overusing opioids is constipation, and therefore it is important to cease or reduce the use of opioids promptly to minimise the side effect of constipation in ICU patients.

Macrogol typically has an onset of action of two to three days; however, it can be used for faecal disimpaction if prescribed in high doses [14]. Despite its established profile, only a small number of patients were prescribed macrogol (four patients in the first cycle, two in the second cycle), suggesting its underutilisation in this protocol. This could be due to patients not being able to tolerate high doses of macrogol orally, specifically those with reduced oral intake or post-operative patients.

Glycerine/bisocodyl suppository, phosphate enema and sodium citrate enema all have a quick onset of action, ranging from two minutes up to 30 minutes. Apart from bisocodyl suppository, the remaining three laxatives are all used for hard, impacted stools as per recommendations by the National Institute for Health and Care Excellence (NICE) Clinical Knowledge summaries [17]. It is documented that this intervention is infrequently used in the bowel protocol, likely because rectal administration is uncomfortable for patients [18].

Naloxegol is an opioid antagonist that is recommended by NICE as an alternative laxative to consider when patients have failed to open their bowels after four days despite other pharmacological measures. It can be given orally once a day. Despite its straightforward administration, it is not commonly prescribed in the ICU as staff reported that they were not familiar with the medication itself [19,20]. Although staff were educated on the use of naloxegol during the educational presentations, no change has been observed between the two cycles. In addition, naloxegol may induce arrhythmia, intestinal perforation and seizure, which is also another reason why this should be used cautiously in ICU settings [21].

Lastly, neostigmine, an acetylcholinesterase inhibitor which encourages intestinal muscular peristalsis, is listed on the protocol to be considered if patients fail to open their bowels for more than 96 hours since admission. Again, due to the lack of awareness of the medication, neostigmine was not recorded to be used at all across the two cycles of observation [22]. Neostigmine is also associated with bradycardia as a known adverse reaction; therefore, caution and monitoring are required when prescribing it [23].

With respect to investigations, daily surgical reviews, including abdominal examination, were conducted by the parent surgical teams for surgical patients. In addition to this, a small number of abdominal CT scans (three scans in the second cycle) were performed throughout the data collection period. It is noted that abdominal CTs remain the definitive imaging of choice to rule out any serious consequences like bowel perforation secondary to severe constipation [24]. The results revealed a poor adherence to performing PR examinations. This is recommended as PR examination is a simple and quick way to detect faecal impaction within the rectum. Low adherence could be due to factors such as the patient's immobility or their post-operative status.

The implementation of small-group educational teaching as an intervention for this audit was deemed informative by the audience, which consisted of a multidisciplinary staff mix including resident doctors, ICU nurses, healthcare assistants, pharmacists, and dieticians. The session was conducted once every week for three weeks, and the estimated staff attendance was around 10 people per session. Although formal feedback following the sessions was not recorded, verbal appraisals were gained from the staff who found the sessions to be informative and effective in raising awareness [25]. Pre- or post-teaching quizzes were not conducted. The information was further re-consolidated by the use of displaying educational posters on the units to remind staff regarding the protocol itself [25]. The posters were placed at each nursing station, near where the computers were set up for ease of attention. They were displayed for three weeks between the two cycles of data collection, and they continued to be kept in the unit following the three-week period for ongoing educational purposes.

Strengths and limitations 

The main strength of this audit is its focus on critical care patients, incorporating cyclical results to guide improvement of bowel protocol adherence. The specific protocol steps are clearly outlined in this study, and the methodology is well defined and can be easily replicated for future re-auditing.

This audit has several limitations. Firstly, this is a single-centre study which took place over a short period of time, with a small number of study subjects. Formal feedback for small-group educational sessions would have provided some valuable insights for identifying any further areas of improvement when delivering the educational sessions.

Secondly, it is important to note that while surgical patients were included in both cycles, due to the nature of operations requiring different post-operative care plans, patients’ bowel management was primarily determined by the parent surgical teams, which limited the use of the bowel protocol. Consequently, no significant differences were observed between the two cycles for this group.

Furthermore, factors such as patients' age, pre-existing co-morbidities, admission illness severity and level of mobility during their ICU stay were not investigated in this audit, yet they could act as confounding variables affecting an individual's bowel motion status. Opioid use could also be evaluated in future audits to determine its impact on bowel motion. A standardised measure, such as morphine milligram equivalents (MME), can be used to quantify opioid exposure. In addition, types of enteral feeding such as nasogastric/nasojejunal (NG/NJ) feeding, full, soft or liquid diet may be assessed in the future as variation in fibre contents can influence bowel motility.

Lastly, patient-level outcome measures were not assessed in this study, which could have provided deeper insights into the clinical significance of managing constipation in critically ill patients.

## Conclusions

Preventing and managing constipation plays a crucial role in aiding the recovery of ICU patients. Simple pharmacological management can be put in place easily to prevent patients from suffering not only discomfort but also to prevent serious complications like bowel perforation, which could potentially lead to mortality. Thus, it is important to have a standardised bowel protocol acting as a practical framework to ensure consistent patient care in the ICU.

The outcome of this audit suggests, but does not confirm, that educational interventions may positively impact the adherence to bowel protocol in ICU patients. For future re-audits, a larger patient sample size, an extended duration of data collection and considerations of the aforementioned confounders may provide more robust insights. Ongoing education on the importance of the bowel protocol may aid better compliance and adherence amongst the clinical staff, which will ultimately improve the quality of care for patients.

## References

[1] de Azevedo RP, Machado FR (2013). Constipation in critically ill patients: much more than we imagine. Rev Bras Ter Intensiva.

[2] Jani B, Marsicano E (2018). Constipation: evaluation and management. Mo Med.

[3] Sharma A, Rao S (2017). Constipation: pathophysiology and current therapeutic approaches. Handb Exp Pharmacol.

[4] Asai T (2007). Constipation: does it increase morbidity and mortality in critically ill patients?. Crit Care Med.

[5] Launey Y, Painvin B, Roquilly A (2021). Factors associated with time to defecate and outcomes in critically ill patients: a prospective, multicentre, observational study. Anaesthesia.

[6] Nassar AP Jr, da Silva FM, de Cleva R (2009). Constipation in intensive care unit: incidence and risk factors. J Crit Care.

[7] Fukuda S, Miyauchi T, Fujita M (2016). Risk factors for late defecation and its association with the outcomes of critically ill patients: a retrospective observational study. J Intensive Care.

[8] Guerra TL, Mendonça SS, Marshall NG (2013). Incidence of constipation in an intensive care unit. Rev Bras Ter Intensiva.

[9] Faucheron JL, Vincent D, Barbut M (2025). How long for gastrointestinal recovery following small bowel, right, or left colonic resection with anastomosis in a full fast-track recovery protocol?. Int J Colorectal Dis.

[10] Rodriguez GM, Gater DR (2022). Neurogenic bowel and management after spinal cord injury: a narrative review. J Pers Med.

[11] Toris GT, Bikis CN, Tsourouflis GS, Theocharis SE (2011). Hepatic encephalopathy: an updated approach from pathogenesis to treatment. Med Sci Monit.

[12] Patel M, Schimpf MO, O'Sullivan DM, LaSala CA (2010). The use of senna with docusate for postoperative constipation after pelvic reconstructive surgery: a randomized, double-blind, placebo-controlled trial. Am J Obstet Gynecol.

[13] Le J, Ji H, Zhou X (2021). Pharmacology, toxicology, and metabolism of sennoside A, a medicinal plant-derived natural compound. Front Pharmacol.

[14] (2025). Factors affecting choice of laxative. https://cks.nice.org.uk/topics/constipation/prescribing-information/factors-affecting-choice-of-laxative/.

[15] Moran BL, Myburgh JA, Scott DA (2022). The complications of opioid use during and post-intensive care admission: a narrative review. Anaesth Intensive Care.

[16] Horn R, Hendrix JM, Kramer J (2025). Postoperative pain control. In: StatPearls [Internet].

[17] (2025). Choice of laxatives. https://cks.nice.org.uk/topics/constipation/prescribing-information/choice-of-laxatives/.

[18] Vyvyan HA, Hanafiah Z (1995). Patients' attitudes to rectal drug administration. Anaesthesia.

[19] Leppert W, Woron J (2016). The role of naloxegol in the management of opioid-induced bowel dysfunction. Therap Adv Gastroenterol.

[20] (2025). Naloxegol for treating opioid‑induced constipation. https://www.nice.org.uk/guidance/ta345.

[21] Rizwan ZM, Garcia R, Mara K, Nei S (2023). Evaluating the safety and efficacy of naloxegol in critically ill opioid-induced constipation patients. Cureus.

[22] Kim TJ, Torres L, Paz A, Lee JS, Park SH, Choi HA, Ko SB (2021). Neostigmine for treating acute colonic pseudo-obstruction in neurocritically ill patients. J Clin Neurol.

[23] Kram B, Greenland M, Grant M, Campbell ME, Wells C, Sommer C (2018). Efficacy and safety of subcutaneous neostigmine for ileus, acute colonic pseudo-obstruction, or refractory constipation. Ann Pharmacother.

[24] Zhang EJ, Toh JW (2023). Severe constipation causing ischaemic stercoral perforation of sigmoid colon. J Surg Case Rep.

[25] Burgess A, van Diggele C, Roberts C, Mellis C (2020). Facilitating small group learning in the health professions. BMC Med Educ.

